# Towards Optimising the Production of and Expression from Polycistronic Vectors in Embryonic Stem Cells

**DOI:** 10.1371/journal.pone.0048668

**Published:** 2012-11-06

**Authors:** Steven Y. Gao, Michelle M. Jack, Christopher O’Neill

**Affiliations:** 1 Centre for Developmental and Regenerative Medicine, Kolling Institute for Medical Research, Sydney Medical School, University of Sydney, Sydney, NSW, Australia; 2 Department of Endocrinology, Royal North Shore Hospital, Sydney, NSW, Australia; University of Massachusetts Medical, United States of America

## Abstract

Polycistronic vectors linked by self-processing 2A peptides have been successfully used in cellular reprogramming. The expression of these vectors has yet to be well documented in embryonic stem cells. In the present study, we generated expression cassettes containing combinatorial arrangements of 3 pancreatic transcriptions factors (*Pdx1, Nkx2.2* and *Ngn3*) together with an *eGFP* reporter, all linked by self-processing 2A peptides. The study tested the utility of constructing complex expression cassettes by ligating multiple components, each flanked by unique restriction sites. This approach allowed flexible and efficient design and construction of a combinatorial array of polycistronic constructs, which were expressed after transient transfection into embryonic stem cells. The inclusion of EGFP provided for a convenient proxy measure of expression and showed that expression was similar regardless of EGFP’s position within a 2A polycistronic construct. Expression of terminal EGFP was 51% and 24% more efficient when linked by T2A compared to F2A or E2A peptides, respectively. The highest level of expression was achieved when all genes in a construct were linked exclusively by T2A peptides. This effect of T2A was independent of the type of promoter used, as a similar increase in terminal EGFP expression was observed when the polycistronic constructs were under the control of a *CAG* promoter compared to the *CMV* promoter, even though the *GAG* promoter was more efficient in this model than the *CMV* promoter. The study provides guidance on design strategies and methods for the efficient generation and expression of 2A polycistronic constructs in embryonic stem cells.

## Introduction

Various approaches have been employed to co-express multiple proteins in cells. These include the use of internal ribosomal entry site (*IRES*) elements [1], dual promoter systems [2] or transfection of multiple vectors [3]. Each of these is associated with a number of limitations such as uneven or unreliable protein expression levels, silencing of some promoters [4], [5], or increased toxicity to cells (with multiple transfections) [6]. An alternative strategy is the use of the self-processing viral 2A peptides, which are reported to allow the efficient expression of multiple genes under the control of a single promoter [7], [8].

The 2A peptides are members of the *cis*-acting-hydrolase elements (CHYSELs) family. These elements were first identified in picornaviruses, and were subsequently found in a number of other viral systems [9]. The most studied 2A peptide sequence is from foot-and-mouth disease virus (F2A) [10]. Other commonly used 2A elements are from the equine rhinitis A virus (E2A) and *Thosea asigna* virus (T2A) [11].

**Table 1:** The sequences of primers used in this study, the restriction enzyme sites used in the study are underlined.

Symbols	Sequences
*eGFP Full F*	*5'-GCCACCATGGTGAGCAAG-3'*
*eGFP Full R*	*5'-TTACTTGTACAGCTCGTCCATGC-3'*
*SacI-GFP-F2A-SacI F*	*5'-GAGCTCGCGGCCGCTGGATCCGCCACCATGGTGAGCAAG-3'*
*SacI-GFP-F2A-SacI R*	*5'-GGATCCAGCGGCCGCGAGCTCGGGCCCTGGGTTGGACTCCACGTCTCCCGCCAACTTGAGAAGGTCAAAATTCAAA* *GTCTGTTTCACCGGACCGCTGCCCTTGTACAGCTCGTCCATGC-3'*
*BamHI/Hind-eGFP*	*5'-GGATCCAAGCTTATGGTGAGCAAGGGCGAG-3'*
*eGFP-SalI*	*5'-GTCGACTTACTTGTACAGCTCGTCCATGC-3'*
*NGN3 Full F*	*5'-ATGGCGCCTCATCCCTTG-3'*
*NGN3 Full R*	*5'-CAAGAAGTCTGAGAACACCAGTGCT-3'*
*BamHI-NGN3-F2A-HindIII F*	*5'-GGATCCGCCACCATGGCGCCTCATCCCTTG-3'*
*BamHI-NGN3-F2A-HindIII R*	*5'-AAGCTTGGGCCCTGGGTTGGACTCCACGTCTCCCGCCAACTTGAGAAGGTCAAAATTCAAAGTCTGTTTCACCGGA* *CCGCTGCCCAAGAAGTCTGAGAACACCAGTGCT-3'*
*BamHI-NGN3-T2A-HindIII R*	*5'-AAGCTTTGGGCCAGGATTCTCCTCGACGTCACCGCATGTTAGCAGACTTCCTCTGCCCTCACCGCTGCCCAAGAA* *GTCTGAGAACACCAGTGCT-3'*
*PDX1 Full F*	*5'-ATGAACAGTGAGGAGCAGTACTACGCG-3'*
*PDX1 Full R*	*5'-CCGGGGTTCCTGCGGTCG-3'*
*SacI-PDX1-E2A-NotI/BamHI F*	*5'-GAGCTCGCCACCATGAACAGTGAGGAGCAGTACTACGCG-3'*
*SacI-PDX1-E2A-NotI/BamHI R*	*5'-GGATCCAGCGGCCGCAGGACCGGGGTTACTTTCAACATCGCCAGCGAGTTTCAACAAAGCGTAGTTAGTACATT* *GGCCAGAACCCCGGGGTTCCTGCGGTCG-3'*
*SacI-PDX1-T2A-NotI/BamHI R*	*5'-GGATCCAGCGGCCGCTGGGCCAGGATTCTCCTCGACGTCACCGCATGTTAGCAGACTTCCTCTGCCCTCGCCA* *GAACCCCGGGGTTCCTGCGGTCG-3'*
*NKX22 Full F*	*5'-CCCCAGTCACAGCCTACATT-3'*
*NKX22 Full R*	*5'-AACAACCGTGGTAAGGATCG-3'*
*NotI-NKX2-T2-BamHIA F*	*5'-GCGGCCGCTGCCACCATGTCGCTGACCAACACAAAGACG-3'*
*NotI-NKX2-T2A-BamHI R*	*5'-GGATCCTGGGCCAGGATTCTCCTCGACGTCACCGCATGTTAGCAGACTTCCTCTGCCCTCGCCAGAACCCCAA* *GTCCACTGCTGGGCC-3'*
*ASEI-CAG F*	*5'-ATTAATAGTAATCAATTACGGGGTCA-3'*
*CAG-NheI R*	*5'-GCTAGCGAATTCTTTGCCAAAATGATGA-3'*
*β-Actin F*	*5'-GCAGGCCTAGTAACCGAGACA-3'*
*β-Actin R*	*5'-AGTTTTGGCGATGGGTGCT-3'*

All commonly used 2A peptides share a highly conserved 18 amino acid region responsible for cleavage. This occurs at the C-terminus of the sequence, between the 2A glycine and 2B proline [11], [12]. The cleavage is achieved through a putative “ribosomal skip” mechanism [13]. The cleavage efficiency of the 2A peptide-linked proteins are generally high, however, it has been demonstrated that the efficiency can be influenced by the identity of the protein at the N-terminus of the peptide [5]. The cleavage efficiency can be improved by placing a Gly-Ser-Gly linker between the N-terminal protein and the 2A peptide [11]. This “flexible” linker creates a space between the N-terminus protein and the peptide, favouring a conformation of the peptide which facilitates efficient cleavage [14].

Self-processing 2A peptides have been used to achieve co-expression of heterogeneous proteins and show cleavage efficiency in a range of differentiated cell lines and embryonic stem cells [4], [7], [8], [11], [15], [16], [17]. Expression of up to five genes linked by 2A sequences under the control of a single promoter has been demonstrated [18], but the efficiency of expression of individual proteins from a polycistronic construct may be lower than their expression from monocistronic constructs [8], although a definitive reason for this reduced efficiency has not been shown.

**Table 2:** Different glycine-serine-glycine (GSG) linkers (Forward) and their corresponding reverse primer sequences (Reverse) used for the generation of 2A polycistronic constructs.

Forward	Reverse
*5'-GGTTCTGGC-3'*	*5'-GCCAGAACC-3'*
*5'-GGCAGCGGT-3'*	*5'-ACCGCTGCC-3'*
*5'-GGAAGAGGA-3'*	*5'-TCCTCTTCC-3'*

The cleavage of 2A peptides may be affected by the nature of the protein attached to its N-terminus [19], [20], the order of the genes used in a specific expression cassette [21], the variant of the 2A peptide used (even if it was derived from the same peptide) [22], and the expression environment [20]. The P2A peptide is reported to have the highest cleavage efficiency in an *in vivo* environment [23], however, there remains relatively limited systematic analysis of the effects of different 2A peptides on the protein expression levels within polycistronic expression cassette. To date, there is not a sufficient body of evidence to allow rational design of 2A-linked polycistronic constructs with optimal expression efficacy.

**Figure 1:**
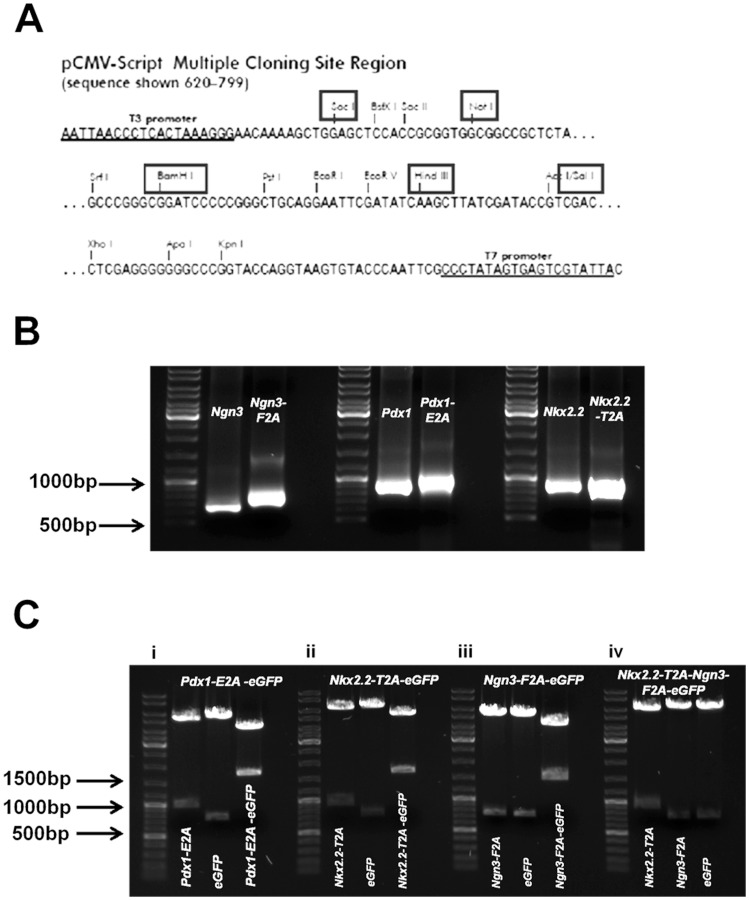
The construction of 2A polycistronic expression cassettes. (**A**) All 2A polycistronic expression cassettes were generated by restriction enzyme digestion and ligation utilising the restriction enzyme cutting sites: SacI, NotI, BamHI, HindIII and SalI (boxed, as described in Materials an methods) present in the multicloning site of the *pCMV-Script* plasmid (Stratagene). (**B**) The amplification of *Ngn3-F2A, Pdx1-E2A and Nkx2.2-T2A*, a size difference between the native full-length gene and the gene with a 2A-peptide tag can be observed using gel electrophoresis. Note that the native *Nkx2.2* amplified from MIN6 cell cDNA contained regions outside of the coding region of the mRNA, as the primers were optimised for maximum efficiency, and thus is larger in size compared to *Nkx2.2-T2A.* Each group of three lanes is (from left to right) molecular weight ladder, native sequence, and native sequence +2A. (**C**) Examples of the flexibility of the restriction enzyme digestion/ligation construction method. Each component of a 2A polycistronic expression cassette can be removed by using the appropriate restriction enzymes: (i) For *Pdx1-E2A-eGFP*, *Pdx1-E2A* can be cut out using SacI and NotI/BamHI (left), *eGFP* can be cut out using NotI/BamHI and SalI (middle) and *Pdx1-E2A-eGFP* can be cut out using SacI and SalI; (ii) for *Nkx2.2-T2A-eGFP*, *Nkx2.2-T2A* can be cut out using NotI and BamHI (left), and *eGFP* can be cut out using BamHI and SalI (middle), and *Nkx2.2-T2A-eGFP* can be cut out using NotI and SalI (right); (iii) for *Ngn3-F2A-eGFP*, *Ngn3-F2A* can be cut out using BamHI and HindIII (left), *eGFP* can be cut out using HindIII and SalI (middle), and *Ngn3-F2A-eGFP* can be cut out using BamHI and SalI (right); (iv) for *Nkx2.2-T2A-Ngn3-F2A-eGFP*, *Nkx2.2-T2A* can be cut out using NotI and BamHI (left), *Ngn3-F2A* can be cut out using BamHI and HindIII (middle), and *eGFP* can be cut out using HindIII and SalI. The sizes of the DNA fragments were confirmed by comparison to the molecular weight ladder on the left of each data set.

The most conventional method for generating polycistronic expression cassettes linked by 2A peptides involves the use of recombinant PCR [24]. This method is relatively inflexible because the use of the same 2A peptide sequence within a PCR results in their self-annealing. Thus, designs are constrained by the need to use either different 2A peptides, or different variables between each linked proteins. Furthermore, any changes made to the structure of the cassette, such as the addition of a gene, requires the re-design of the large primers used in construction as well as the cost and time-consuming process of re-constructing the cassette. These matters limit the feasibility of constructing large numbers of constructs for screening and optimisation purposes. The present study assesses a more flexible approach for generating 2A polycistronic expression cassettes. Each gene and its assigned 2A peptide were designed to have flanking restriction enzyme sites. This allowed the generation of many different gene combinations by adding and removing gene-2A peptide units using standard restriction enzyme digestion and ligation methodologies. This approach allows the use of multiple copies of a given 2A peptide within an expression cassette.

The aim of this study was to systematically analyze the effects of design variables on the efficiency of production and expression of 2A polycistronic vectors in mouse embryonic stem cells. The number of genes, their order within a construct, the type of promoter and the type of 2A peptide used were examined. We found that the type of promoter and the 2A peptides used independently impacted on the efficiency of expression and demonstrated the utility and efficiency of the this approach to testing polycistronic constructs.

**Table 3:** List of vectors generated for this study.

Vectors	
*pCMV-eGFP*	*CMV-G*
*pCMV-Ngn3-F2A-eGFP*	*CMV-N-F2A-G*
*pCMV-Pdx1-E2A-eGFP*	*CMV-P-E2A-G*
*pCMV-Nkx2.2-T2A-eGFP*	*CMV-N2-T2A-G*
*pCMV-Pdx1-E2A-Ngn3-F2A-eGFP*	*CMV-P-E2A-N-F2A-G*
*pCMV-Pdx1-E2A-Nkx2.2-T2A-eGFP*	*CMV-P-E2A-N2-T2A-G*
*pCMV-Nkx2.2-T2A-Ngn3-F2A-eGFP*	*CMV-N2-T2A-N-F2A-G*
*pCMV-Pdx1-E2A-Nkx2.2-T2A-Ngn3-F2A-eGFP*	*CMV-P-E2A-N2-T2A-N-F2A-G*
*pCMV-eGFP-F2A-Pdx1-E2A-Nkx2.2-T2A-Ngn3*	*CMV-G-F2A-P-E2A-N2-T2A-N*
*pCMV-Ngn3-T2A-eGFP*	*CMV-N-T2A-G*
*pCMV-Pdx1-T2A-eGFP*	*CMV-P-T2A-G*
*pCMV-Pdx1-T2A-Ngn3-F2A-eGFP*	*CMV-P-T2A-N-F2A-G*
*pCMV-Pdx1-E2A-Ngn3-T2A-eGFP*	*CMV-P-E2A-N-T2A-G*
*pCMV-Pdx1-T2A-Nkx2.2-T2A-Ngn3-T2A-eGFP*	*CMV-P-T2A-N2-T2A-N-T2A-G*
*pCAG-Pdx1-E2A-Nkx2.2-T2A-Ngn3-F2A-eGFP*	*CAG-P-E2A-N2-T2A-N-F2A-G*
*pCMV-Pdx1-T2A-Nkx2.2-T2A-Ngn3-T2A-eGFP*	*CMV-P-T2A-N2-T2A-N-T2A-G*

## Materials and Methods

### Cell Cultures

The mouse ES cell line D3 (P22-P32) was maintained on 0.1% (v/v) gelatin (BDH Chemicals, Poole, UK) coated tissue culture dishes in ES cell media (DMEM (Thermo Fisher Scientific, Scoresby, VIC, Australia) supplemented with 10% (v/v) FBS (Invitrogen, Carlsbad, CA), 0.1 mM β-mercaptoethanol (Sigma, St. Louis, MO), 50 µg/mL penicillin/streptomycin mix (Sigma, St. Louis, MO) and 1000 units of Leukaemia inhibitory factor (LIF) (GIBCO, Grand Island, NY)). ES cell media was changed every 1–2 days and the cells were passaged using 0.25% (w/v) trypsin-EDTA (GIBCO) upon reaching 70% confluence. Cells were cultured in 5% CO_2_ in air at 37°C.

**Figure 2:**
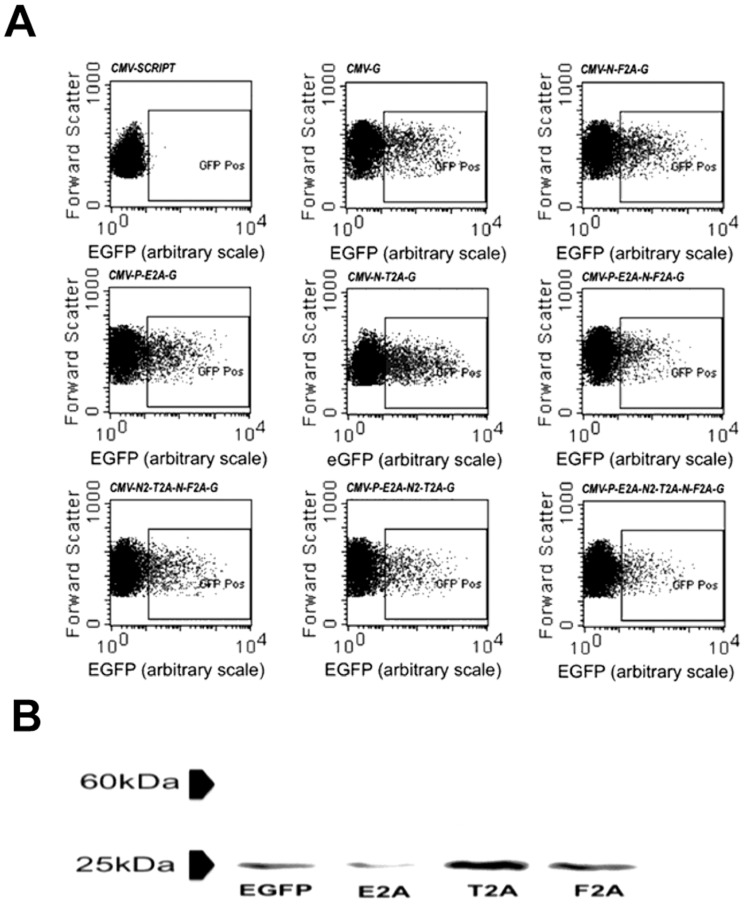
Analysis of expression and cleavage of polycistronic vectors in mouse ES cells. (**A**) Each of the vectors shown in **Table 3** were transiently transfected into mouse ES cells and EGFP expression assessed 24 h later. Representative analysis of at least 3 replicates is shown for each vector. The *pCMV-Script* backbone plasmid was used as a negative control and expression above these levels were taken as an evidence of positive EGFP expression. Each graph shows the relative level of EGFP expression in arbitrary units per cell against forward scatter. (**B**) The cleavage efficiency of E2A, T2A and F2A in mouse ES cells was determined by using western blot analysis of EGFP from protein samples extracted from cells transfected with two-gene vectors each linked by one of the three 2A peptides.

**Figure 3:**
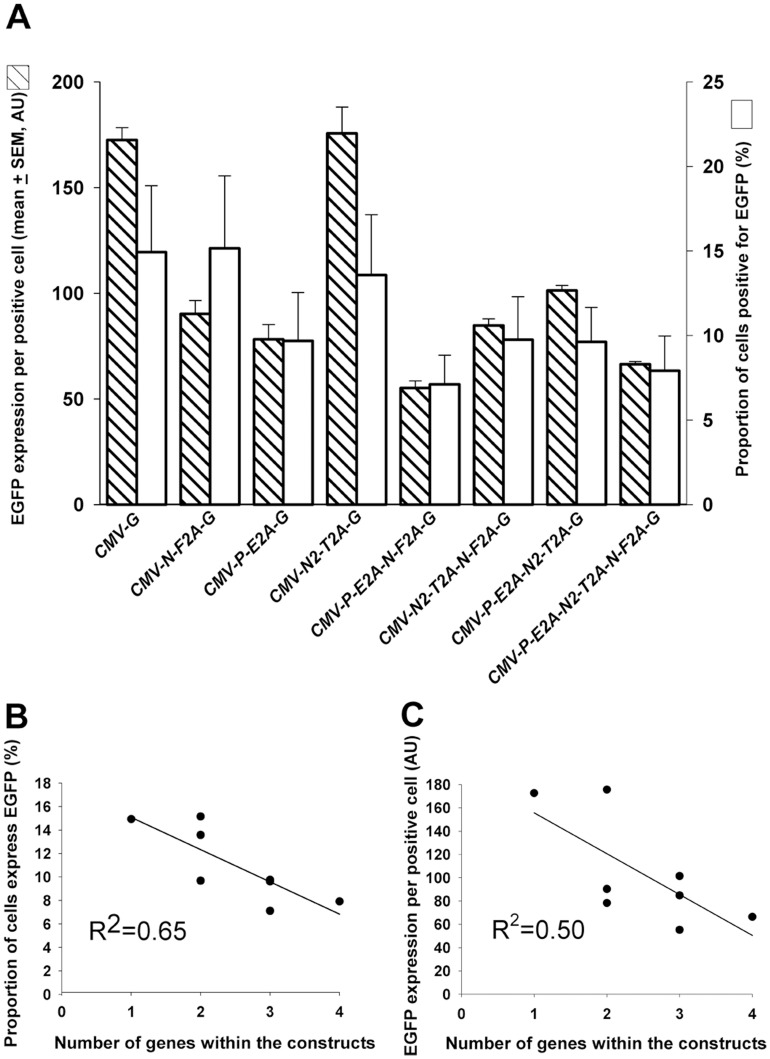
Analysis of EGFP expressing mouse ES cells 24 **h post transfection.** (**A**) Comparative analysis was conducted to determine the relationship between the mean EGFP expression per positive cell () and the proportion of cells expressing EGFP (). Data presented as mean ± SEM from 3 independent experiments. AU = arbitrary units. (**B**) The relationship between the proportion of cells expressing EGFP and number of genes contained within the constructs. Each point on the graph depicts the mean percentage of EGFP expressing cells transfected by one type of vector. Data was obtained from 3 independent experiments. (**C**) The relationship between EGFP expression per cell and number of genes within constructs. An inverse linear relationship between the number of genes contained within each vector and the mean EGFP expression levels per positive cell was observed, within an increased number of genes associated with decreased EGFP expression levels. R^2^ = linear regression coefficient. AU = arbitrary units.

### Amplification of the Full-length Sequences Using PCR

The primers used for generating constructs, and their restriction sites are shown in (Table 1). Full-length sequences of mouse *Ngn3* and *Pdx1* were amplified from *pAd-Ngn3-I-nGFP* and *pAd-Pdx1-I-nGFP* plasmids, respectively (plasmid number 19410 & 19411, kindly supplied by Dr. D. Melton through Addgene, Cambridge, MA) [25] using primers *Pdx1 Full F, Pdx1 Full R, Ngn3 Full F and Ngn3 Full R* (**Table 1**). The full-length sequence of *Nkx2.2* was amplified using the mouse insulinoma (MIN6) cell line [26] cDNA as a template with primers *Nkx22 Full F* and *Nkx22 Full R* (**Table 1**). The full-length sequence of *eGFP* was amplified from the 613bp to 1329bp of the *pEGFP-C1* plasmid (Clontech, Mountain View, CA) using primers *eGFP Full F* and *eGFP Full R* (**Table 1**).

**Figure 4:**
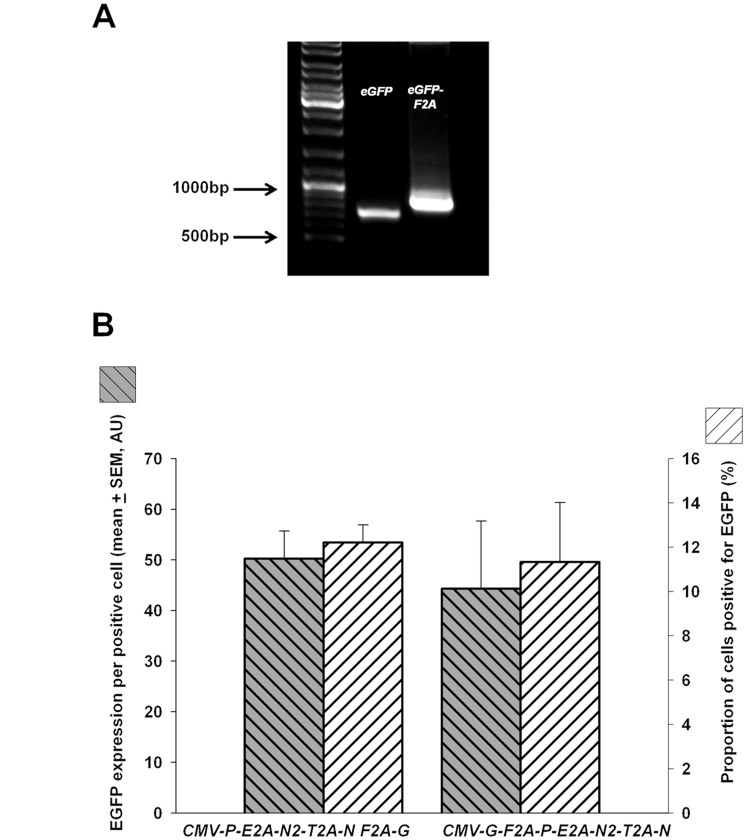
The expression of a gene at different locations within a 2A polycistronic expression cassette. (**A**) *eGFP-F2A* was amplified using PCR, and the difference in size compared to the native *eGFP* can be observed using gel electrophoresis. (**B**)The expression of EGFP was assessed when it was moved from the final position to the first position withinin a four-gene polycistronic construct. No statistical difference in EGFP expression was found (left axis), and no difference was found in the proportion of the cells expressing EGFP 24 hr after the cells were transfected with the two constructs (right axis). Data presented as mean + SEM from 3 independent experiments. AU  =  arbitrary units.

### Vector Construction

The stop codons of *Pdx1, Nkx2.2* and *Ngn3* were removed from their respective reverse primers and a sequence encoding a glycine-serine-glycine (*GSG*) linker (**Table 2**) was added to the 3'-end of each gene. This linker is reported to facilitate efficient cleavage of the 2A peptides [27]. *PCMV-Script* (Stratagene, Cedar Creek, TX) was used as the backbone plasmid. Each gene was assigned a 2A peptide and flanked with appropriate restriction enzyme sites as found on the *pCMV-Script* plasmid. The following sequences were generated for the initial testing of the expression cassettes: *SacI-Pdx1-E2A-NotI/BamHI, NotI-Nkx2.2-T2A-BamHI, BamHI-Ngn3-F2A-HindIII, BamHI/HindIII-eGFP-SalI.* To move the *eGFP* from the final position to the first position, the *F2A* sequence attached to *Ngn3* was removed and a stop codon was added to the end of *Ngn3* to generate *BamHI-Ngn3-HindIII.* This replaced *BamHI-Ngn3-F2A-HindIII*. The stop codon of the *eGFP* was removed and the *F2A* sequence was added to the 3′-end of the *eGFP* to become *SacI-eGFP-F2A-SacI*. *T2A* sequences were used in place of the *F2A* and *E2A* sequences, while no changes to the restriction enzyme sites used were made.

The template sequences of the *F2A*, *E2A* and *T2A* peptides were obtained from a previously published study [11]. The primers used to generate the 2A constructs were: *BamHI/HindIII-eGFP, eGFP-SalI, BamHI-Ngn3-F2A-HindIII F, BamHI-Ngn3-F2A-HindIII R, SacI*-*Pdx1-E2A-NotI/BamHI F, SacI*-*Pdx1-E2A-NotI/BamHI R, NotI-Nkx2-T2A-BamHI F, NotI-Nkx2-T2A-BamHI R, SacI-eGFP-F2A-SacI F, SacI-eGFP-F2A-SacI R* (**Table 1**). The amplified sequences were then ligated into the multicloning site of *pCMV-Script* using T4 ligase (Promega, Madison, WI) to obtain the desired 2A polycistronic vectors. All restriction enzymes and buffers used were purchased from New England Biolabs (Ipswich, MA).

The primers used for the T2A experiments are*: BamHI-Ngn3-T2A-HindIII F*, *BamHI*-*Ngn3-T2A-HindIII R* and *SacI*-*Pdx1-E2A-NotI/BamHI F*, *SacI*-*Pdx1-T2A-NotI/BamHI R* (**Table 1**). The generated sequences were then ligated into the desired locations of the 2A polycistronic vectors to replace the original sequences.

**Figure 5:**
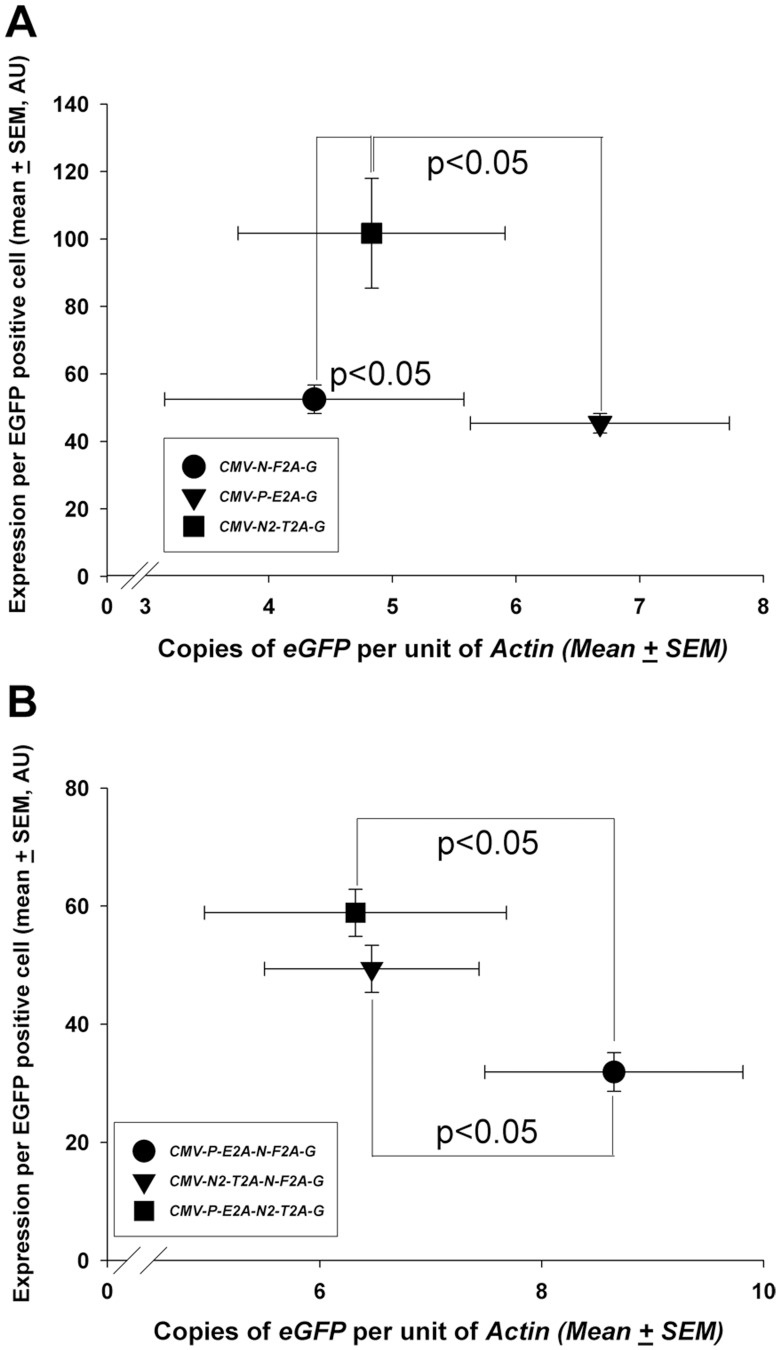
Analysis of vector DNA copy numbers using qPCR. Difference in EGFP expression in mouse ES cells transfected with two-gene and three-gene vectors compared to vector DNA copy numbers in the transfected cells. Vector DNA copy number was determined using qPCR and normalised to *Actin*. Even though significant differences in the mean EGFP expression per positive cell was observed between T2A containing constructs and non-T2A containing constructs for both two-gene (**A**) and three-gene (**B**) polycistronic vectors, the increased EGFP expression was not correlated with high vector DNA copy number. Data presented as mean ± SEM from 3 independent experiments. AU  =  arbitrary units.

**Figure 6:**
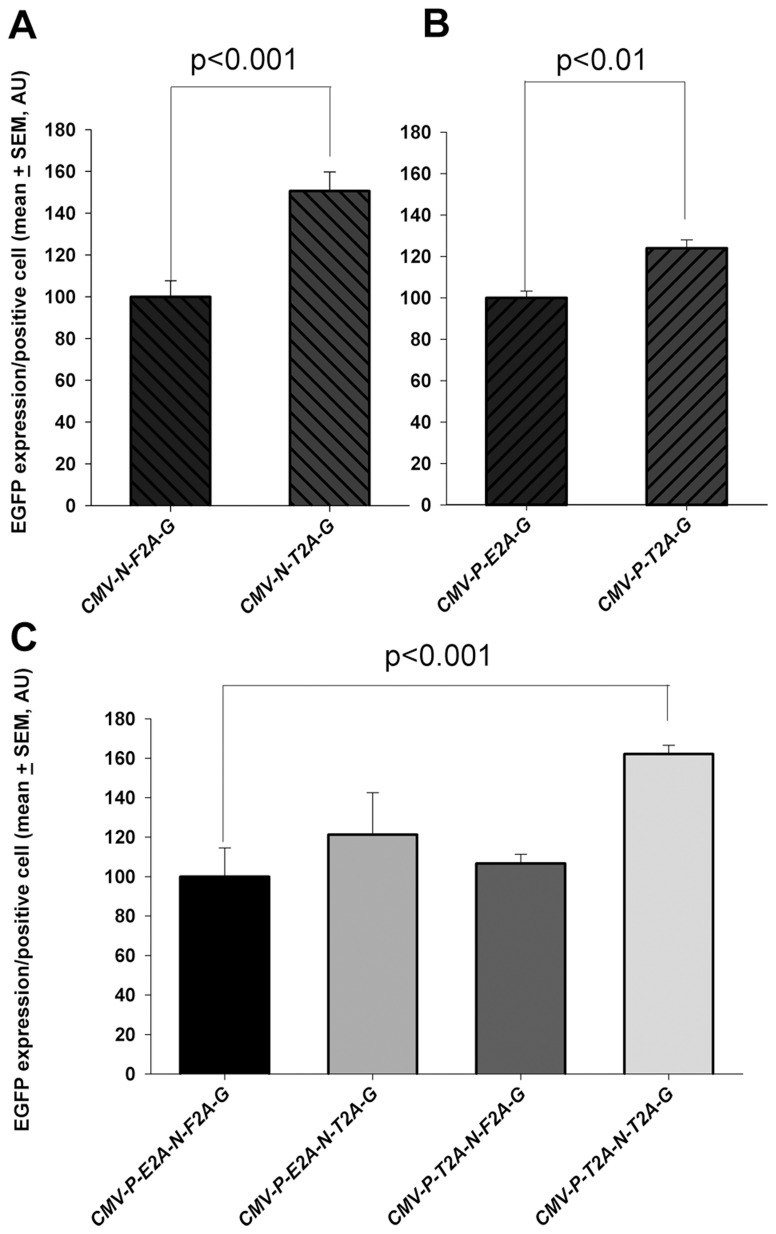
Analysis of the difference between *T2A, E2A* and *F2A* on EGFP expression by cells. (**A**) Replacing *F2A* peptide with *T2A* peptide in a two-gene construct caused a significant increase in mean EGFP expression per positive cell. (**B**) Replacing *E2A* with *T2A* in a two-gene construct caused a significant increase in the mean EGFP expression per positive cell. (**C**) In a three-gene construct, the expression of EGFP was not altered after replacing either a single single F2A or E2A with T2A. A significant increase in EGFP expression was detected when both F2A and E2A were replaced with T2A. Data presented as mean ± SEM from 3 independent experiments. AU  =  arbitrary units.

### Transient Transfection of Constructs

Mouse ES cells were trypsinised and plated at 100,000 cells/cm^2^ on 48 well tissue culture plates (Nunc, Roskilde, Demark). Transfection media was ES cell media without penicillin/streptomycin. Sterile vector DNA at a concentration of 1.2 µg/100,000 cells was transfected using Lipofectamine 2000 at 3.6 µL/1.2 µg of DNA (Invitrogen). Samples were analysed 24 h post transfection. The backbone *pCMV-Script* plasmid (Stratagene) was used as a negative control.

### Cloning of the CMV Early Enhancer/chicken β-actin (*CAG*) Promoter

In order to determine whether the choice of promoter influenced the efficiency of *T2A*, the *CMV* promoter was replaced by a *CAG* prompter/enhancer. The full *CAG* promoter sequence with a 5'-*NheI* restriction enzyme overhang was amplified with primers *ASEI*-*CAG F* and *CAG NheI R* (**Table 1**) from plasmid *pCAGEN* (kindly provided by Dr. C. Cepko through Addgene, plasmid number 11160) [28] using PCR. The *CMV* enhancer/promoter from the *pCMV-Pdx1-Nkx2.2-Ngn3-eGFP* vectors was removed by digesting the vector with AseI and NheI. The *CAG-NheI* sequence was then digested and ligated into the AseI and NheI sites of the *Pdx1-Nkx2.2-Ngn3-eGFP* vectors.

**Figure 7:**
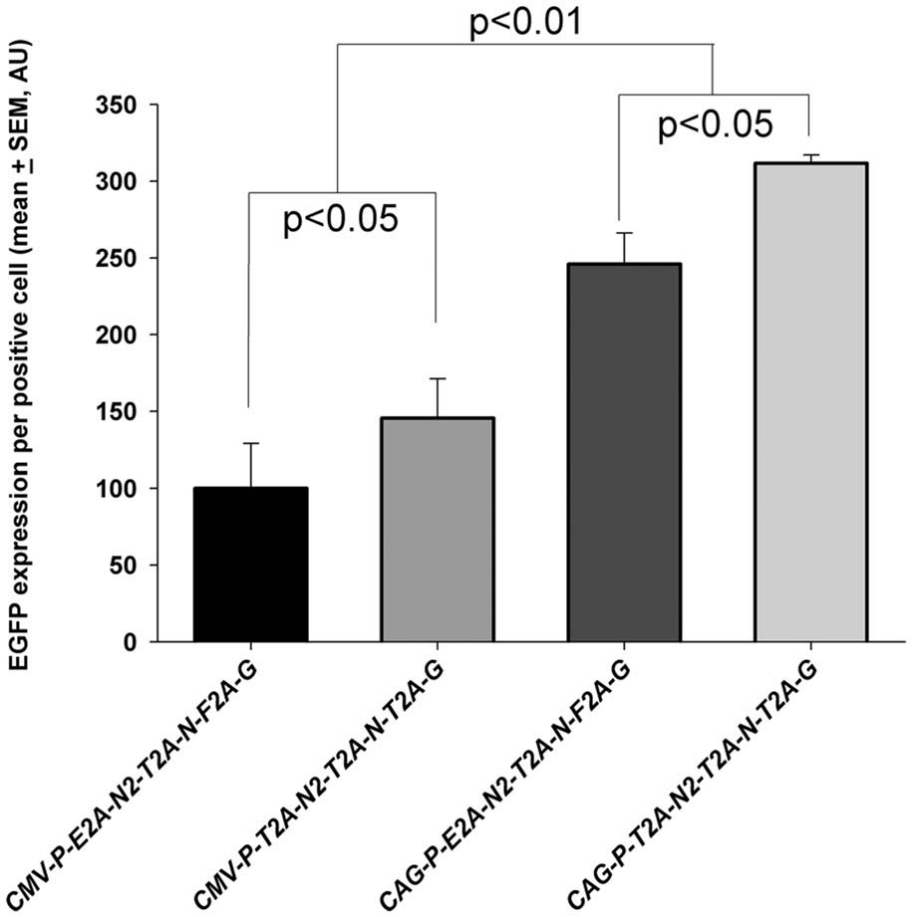
The effect of a different promoter on the beneficial effect of *T2A* on EGFP expression in four-gene constructs. A significant increase (P<0.05) in mean EGFP expression per positive cells can be observed when comparing a four-gene construct linked with only T2A peptides and a four-gene construct linked with a mixture of E2A, T2A and F2A peptides, irrespective of promoter. The *CAG* promoter in place of the *CMV* promoter increased expression of EGFP, irrespective of the 2A sequences (p<0.01). There was a significant interaction effect between 2A sequence and promoter (p<0.05), showing that CAG together with al T2A gave the highest level of expression. Data presented as mean + SEM from 3 independent experiments. AU  =  arbitrary units.

### Fluorescence-activated Cell Sorting

Transfected cells were analyzed 24 h post transfection using a BD DACS Calibur flow cytometer (BD Biosciences, Franklin Lakes, NJ). The cells were gated based on size and EGFP expression was detected at an excitation frequency of 488nm. The final data was analysed using the BD Cell Quest Pro software (BD Biosciences).

### Determining Vector DNA Copy Numbers

In order to analyse the vector copy number in the transfected mouse ES cells, DNA from the cells was extracted using the Wizard Genomic isolation System (Promega) following the manufacturer’s instructions. An equal amount of DNA from each sample was added to each quantitative PCR (qPCR) reaction. The qPCR primer sequences for *eGFP* were obtained from a previous study [29]. *Beta-Actin* was used as a control (**Table 1**). All samples were then amplified using a Rotorgene RG3000 (Corbett Life Science, Concord, NSW, Australia). The Ct value of the *eGFP* was normalised against its corresponding *β-Actin* Ct value. The results are represented as the “units of *eGFP* DNA per unit of *Actin*”. This provides a measure of relative transfection efficiency.

### Western Blot

To investigate the cleavage efficiency of each of the 2A peptides used in the study, protein samples were extracted from an equal number of cells transfected with *Pdx1-E2A-eGFP*, *Nkx2.2-T2A-eGFP* and *Ngn3-F2A-eGFP* constructs. Constructs were separated by 12% (w/v) SDS-PAGE and transferred onto nitrocellulose membranes. The membranes were blocked using 5% skim milk in TBS-Tween (TBST, Tris-Buffered Saline with 0.05% v/v Tween 20) before incubation with GFP primary antibody (1∶5000) (Abcam, Cambridge, MA, USA) overnight at 4°C and horseradish peroxide conjugated secondary antibody (1∶5000) (Abcam) for 1 h at room temperature. The samples were developed using SuperSignal West Femto Chemiluminescent Substrate (Thermo Fisher Scientific, Scoresby, VIC, Australia) and visualised using chemiluminescence on a Fujifilm LAS-4000 (GE Healthcare, Waukesha, WI).

### Statistical Analysis

Statistical analysis was conducted using SPSS statistics software (IBM, Armonk, NY). Students T-tests were conducted to compare the difference in EGFP expression level between each transfected sample and vector DNA copy numbers between *T2A* and non-*T2A* containing vectors. Univariate analysis was conducted to determine the effect of different construct designs containing *T2A* on the expression levels of EGFP when *eGFP* was the terminal gene within the construct. The difference between results was considered significant if p<0.05.

## Results

The present study constructed a range of polycistronic expression cassettes by linking a number of *Gene-2A* sequences using unique restriction enzyme sites. This contrasts with the conventional recombinant PCR method for generating 2A polycistronic expression cassettes [24]. The method made use of the multicloning site of the *pCMV-Script* plasmid (**Figure 1A**). A 2A peptide was assigned to each gene (F2A was assigned to *Ngn3*, E2A to *Pdx1* and T2A to *Nkx2.2*) and amplified using conventional PCR. The size difference between the native gene and the gene with a 2A tag can be visualised using gel electrophoresis (**Figure 1B**). This approach allowed each component within a 2A polycistronic expression cassette to be edited by conventional restriction digestion/ligation (**Figure 1C**).

All vectors generated in the study can be found in **Table 3**. Gene expression from the generated vectors was first screened using FACS detection of the expression of EGFP protein, which was incorporated as the last gene within each of the constructs. The cells transfected with the *pCMV-Script* backbone plasmid were used as negative controls. An equal amount of each vector was used to transfect mouse ES cells in all samples and EGFP expression was assessed 24 h post transfection by FACS analysis. All vectors generated using the restriction enzyme digestion/ligation method resulted in the expression of EGFP in a proportion of the transfected population (**Figure 2A**). Western blot analysis of EGFP was performed on cells transfected with one of three different two-gene constructs (**Figure 2B**). It was expected that if cleavage was incomplete a chimeric protein of larger molecular mass would result. In each case, all of the EGFP detected was of the expected size, showing efficient cleavage by all three 2A sequences joined by the restriction enzyme method. These results show that each of the 2A sequences tested had equally high cleavage efficiencies and that the measurement of EGFP was a reliable proxy for assessing vector expression and 2A cleavage efficiency in this model.

No relationship was found between the mean EGFP expression per positive cell and the proportion of cells positive for EGFP (**Figure 3A**). There was an inverse linear relationship (R^2^ = 0.65) between the number of genes contained within each vector and the proportion of cells that expressed detectable EGFP (**Figure 3B**), and a similar relationship with the level of expression in those cells that were positive for EGFP signal (R^2^ = 0.50) (**Figure 3C**).

In order to investigate whether the position of a gene located in a 2A polycistronic expression cassette has an impact on its protein expression. An *eGFP-F2A* sequence was amplified using PCR (**Figure 4A**) and ligated into the first position of a four-gene expression cassette. Moving the *eGFP* from the last to first position within the construct did not change the level of EGFP expressed (**Figure 4B**), and confirms the utility of the flanking restriction enzyme approach for modifying the constructs.

As well as the inverse relationship between expression and the number of genes within a construct, there was also marked variability in EGFP expression between different two and three-gene constructs. To assess whether this was accounted for by differences in transfection efficiency, the *eGFP* copy number (relative to *β-actin*) was measured by qPCR and compared to the level of EGFP expression. This showed that the level of EGFP expression did not show an obvious relationship to the gene copy number. It was observed that a construct containing the T2A peptide had the highest level of expression relative to copy number (Figure 5A, 5B). The constructs tested, however, contained a range of genes so it was not possible to determine whether the observed variability was accounted for by the efficiency of transcription/translation of the different genes or due to the presence of different 2A sequences. We therefore used the flanking restriction enzyme approach to cassette re-design to change the constructs to have all genes linked by T2A. Thus, *Pdx1-T2A* and *Ngn3-T2A* were used to directly replace *Pdx1-E2A* and *Ngn3-F2A* in the original cassettes. The expression of these new vectors in ES cells showed that in otherwise identical two-gene cassettes the use of a T2A-linker increased expression compared to F2A or E2A (**Figure 6A**). Using this same approach to reconstruction of the cassette in three-gene constructs, replacing only one 2A peptide (E2A or F2A) with T2A did not result in a significant change in EGFP expression, but exclusive use of *T2A* in this three-gene vector resulted in an approximate 60% increase in EGFP expression (**Figure 6B**).

*CMV* is not always an efficient promoter in ES cells and it was therefore of interest to determine whether the increased efficiency of T2A was also manifested when the construct was under a stronger promoter. We re-designed four-gene cassettes to be expressed under either *CMV* or the *CAG* enhancer/promoter. Constructs were designed to have either different 2A sequences between each gene or linked exclusively by T2A sequences. EGFP expression was significantly higher under the *CAG* promoter than *CMV* vectors, irrespective of the 2A sequences used (including those linked exclusively with T2A) (**Figure 7**). The result shows that the beneficial effect of T2A sequence on expression was independent of the efficiency of the promoter used.

## Discussion

The present study constructed various polycistronic expression cassettes by linking a number of *Gene-2A* sequences, each flanked by unique restriction enzyme sites. This method allowed the generation of a combinatorial array of constructs containing up to four genes. This provides for a more time and cost-effective approach to the construction of multiple constructs and provides for a rapid method of their re-engineering as required [24]. Additionally, this method allowed the use of multiple copies of the same 2A peptide within an expression cassette without the need for use of sequence variants [30]. This eliminated any potential changes in expression or cleavage efficiency caused by using sequence variants of the same peptide [22], and therefore reduces the level of validation of the constructs required.

This study confirms several reports that the efficiency of transcription decreases with an increase in the number of genes contained in a 2A polycistronic vector [7], [8]. We found that a significant component of this loss of efficiency could be ameliorated by changing the identity of the 2A sequence used. Expression cassettes containing the *T2A* sequence were transcribed more efficiently than those containing either *F2A* or *E2A* in mouse ES cells, and four-gene cassettes linked exclusively with T2A peptide had higher expression levels than the same genes linked by a mix of 2A sequences.

It is reported that the *CMV* promoter is not consistently effective in ES cells [31], [32], [33], [34], so we were interested to examine whether the apparent beneficial effect of the T2A peptide was also manifested under the control of a stronger promoter. The *CAG* promoter/enhancer is reported to act efficiently in ES cells [35] and we found a range of construct designs were more efficiently expressed under the control of the *CAG* promoter compared with *CMV*. Despite this increased efficiency there was still a further beneficial effect of the T2A peptide.

It is been well documented that similar levels of gene expression can be achieved for all genes within a 2A polycistronic vector [4], [8], [11], [17], [18], [36], therefore, it is reasonable to assume that the expression of EGFP is representative of the expression levels of all the proteins within a single expression cassette. The advantage of using a reporter like EGFP for this study is that it provides a relatively cost effective method for detecting and quantifying protein expression levels and isolation of transfected cells by FACS.

There are conflicting reports on the relative cleavage efficiency of the 2A sequences. Some reports show a greater efficiency of T2A compared to F2A and E2A [19], [23] and another that F2A was better than T2A and E2A [11]. In those studies GSG linkers on the N-terminus of the 2A peptides were not included in the design. This linker can facilitate almost complete cleavage of the peptides [15], [37], and in this study we found that each of the three 2A peptides used had an equally high cleavage efficiency in mouse ES cells. The observation that EGFP production was the same whether it was first or last in the sequence of an expression cassette provides confirmation that in this model cleavage efficiency was not a limiting factor to efficiency of expression. The results show that the identity of the 2A sequences was a primary determinant of transcription and/or translation of the polycistronic expression cassettes. The mechanisms involved require further investigation and their understanding may provide further insights into strategies of optimisation of gene expression. The P2A sequence was recently shown [23] to have high cleavage efficiency in a polycistronic expression system *in vivo*, further illustrating the need for continued exploration of these options.

This study demonstrates the improved utility and efficiency of a flanking restriction enzyme based construction approach for 2A polycistronic expression cassettes for the generation of a combinatorial range of vectors. It enabled rapid analysis of the effects of a range of factors on the expression efficiency of various designs. This allowed the identification that the 2A sequence was a major determinant of the efficiency of expression of these vectors, and provides for a convenient method of use of the same 2A sequence to link multiple genes. The study points to the need for careful analysis of this aspect of design for each use. Further analysis is required to assess whether this will apply to all target cells and all expressed genes. The results provide an efficient platform for the rapid development and testing of polycistronic constructs for use in the budgeoning field of cellular reprogramming.
